# Methanolic plant extracts: emerging biotherapeutic alternatives for animal wound infection control

**DOI:** 10.3389/fvets.2026.1812558

**Published:** 2026-04-13

**Authors:** Safia Arbab, Hanif Ullah, Khalid J. Alzahrani, Khalaf F. Alsharif, Fuad M. Alzahrani, Abdulwahab Abuderman, Zhongyi Zeng

**Affiliations:** 1 Medicine and Engineering Interdisciplinary Research Laboratory of Nursing & Materials, Nursing Key Laboratory of Sichuan Province, West China Hospital, West China School of Nursing, Sichuan University, Chengdu, Sichuan, China; 2 Lanzhou Institute of Husbandry and Pharmaceutical Sciences, Chinese Academy of Agricultural Sciences, Lanzhou, China; 3 Department of Clinical Laboratories Sciences, College of Applied Medical Sciences, Taif University, Taif, Saudi Arabia; 4 Basic Medical Sciences Department, College of Medicine, Prince Sattam Bin Abdulaziz University, Al-Kharj, Saudi Arabia; 5 Outpatient Department, West China Hospital of Sichuan University, West China School of Nursing, Sichuan University, Chengdu, China

**Keywords:** antibacterial activity, bacterial isolates, ciprofloxacin, medicinal plants, methanol extract, wound infection

## Abstract

Wound infections caused by bacterial pathogens remain a major health concern, often delaying tissue repair and increasing morbidity. This study evaluated the phytochemical composition and antibacterial activity of methanolic leaf extracts from *Aloe vera, Lawsonia inermis, Azadirachta indica, Curcuma longa*, and *Achyranthes aspera* as potential natural alternatives for managing wound-associated infections. Phytochemical screening revealed the presence of alkaloids, flavonoids, tannins, phenolics, saponins, and steroids in all extracts, with *L. inermis* and *C. longa* showing particularly high levels of phenolics, tannins, and flavonoids, while *A. Indica* exhibited abundant alkaloids and saponins. From 123 wound samples, six bacterial species were isolated: *Staphylococcus aureus* (38; 30.9%), *Streptococcus agalactiae* (17; 13.8%), *Proteus mirabilis* (22; 17.9%), *Escherichia coli* (18; 14.6%), *Pseudomonas aeruginosa* (15; 12.2%), and *Klebsiella pneumoniae* (13; 10.6%). Antibacterial activity assessed using the agar well diffusion method showed that *A. indica* produced the highest inhibition zones (12.5 ± 0.5 mm to 20.4 ± 0.9 mm), followed by *C. longa* (11.6 ± 0.5 mm to 19.2 ± 0.7 mm). The positive control, ciprofloxacin (10 μg), exhibited inhibition zones ranging from 20.8 ± 0.5 mm to 26.1 ± 0.4 mm, whereas the negative control (10% DMSO) produced no inhibition. The observed antibacterial activity is likely due to the synergistic effects of bioactive phytochemicals, which disrupt bacterial cell membranes and inhibit bacterial growth. These findings indicate that *A. indica* and *C. longa* possess strong antibacterial potential and may serve as promising natural agents for managing wound infections caused by clinically relevant pathogens. Further *in vivo* and toxicity studies are recommended to validate their therapeutic applicability.

## 1 Introduction

Wound infections still account for a significant portion of the world's health expenditure, frequently leading to delayed recovery, extended hospitalization, and higher healthcare expenses. The typical pathogenic bacteria associated with wound sepsis include *Staphylococcus aureus, Escherichia coli, Pseudomonas aeruginosa*, and *Klebsiella pneumoniae*. These pathogens have also developed multidrug-resistant strains, which have only complicated treatment, and finding an effective alternative is urgently needed (1, 2).

Plants have been used in traditional medicine as medicines to treat wounds and related infections. The therapeutic potential of them stems mostly from their bioactive secondary metabolites, such as alkaloids, flavonoids, tannins, terpenoids, and phenolics, which have antibacterial, antioxidant, anti-inflammatory, and wound-healing effects (3, 4). Phytochemical studies, especially those involving methanol extracts, are popular because of the range of bioactive substances they recover (5, 6).

*Aloe vera* (L.) is popular among medicinal plants as it is used in wound management, burns, and skin infections. It includes anthraquinones, flavonoids, and saponins that not only suppress microbial growth but also stimulate collagen formation and fibroblast proliferation, thereby accelerating wound healing (7). Similarly, *Lawsonia inermis (henna)* has been used conventionally as a topical dressing on skin ailments and injuries. The active naphthoquinone derivative, lawsone, is a powerful antibacterial and antifungal agent, which is one of the factors in its therapeutic effect (8, 9).

Another medicinal plant that is extensively used is *Azadirachta indica* (neem), which is a broad-spectrum antimicrobial, anti-inflammatory, and immunomodulatory plant. Neem leaf extracts have been shown in both experimental and clinical research to be of great value in terms of antibacterial activity and wound *contraction* (10, 11). Curcuma longa (turmeric) is a polyphenolic compound (curcumin) that is widely used in Ayurveda and modern herbal products due to its antibacterial, antioxidant, and anti-inflammatory properties. Curcumin promotes wound healing by lowering the load of microbes, inducing angiogenesis, and regulating inflammatory processes (12, 13).

Moreover, Achyranthes aspera (prickly chaff flower) has been used in traditional medicine to treat wounds, skin infections, and inflammatory disorders. Its leaf extracts containing methanol have been established to have antimicrobial, antioxidant, and anti-inflammatory properties, which are essential in wound healing (14, 15).

Since the issue of antibiotic resistance is escalating, these plants will be good potential sources of plant-based antimicrobial formulations. Consequently, the purpose of the current research is to explore the antibacterial effects of methanol leaf extracts of *A. vera, L. inermis, A. indica, C. longa*, and *A. aspera* in relation to wound infection-causing bacteria to confirm their future use as a natural alternative in wound management.

Thus, the purpose of the study was to assess the phytochemical content of methanolic leaf extracts of *Aloe vera, Lawsonia inermis, Azadirachta indica, Curcuma longa*, and *Achyranthes* and determine their antibacterial activity by the agar well diffusion technique against the major bacteria infecting wounds. The results are meant to offer scientific support to the conventional use of these plants and to determine the possible natural substitutes for the use of these plants in the management of wound infection.

## 2 Methods

### 2.1 Ethics statement

All animal experiments were carried out in accordance with the regulations regarding the use of animals in toxicology. The research focused on the Antibacterial Activity of Methanol Leaf Extracts of Medicinal Plants: A Natural Alternative Against Wound Infection Bacteria. The Animal Administration and Ethics Committee of the Chinese Academy of Agricultural Sciences' Lanzhou Institute of Husbandry and Pharmaceutical Sciences approved all the experiments.

### 2.2 Sample collection and study design setting

A total of 123 wound isolates were collected from infected livestock, including cattle, goats, and sheep. Wound exudates were obtained using sterile cotton swabs from infected areas of each animal and transferred to freshly prepared nutrient agar and mannitol salt agar (Oxoid) slants. Following standard bacteriological procedures, the samples were cultured on various media, including mannitol salt agar, 5% sheep blood agar, and chocolate agar. The cultures were then incubated at 37 °C for 24–48 h (6).

### 2.3 Medicinal plants collection

Fresh and healthy leaves of five medicinal plants, *Aloe vera, Lawsonia inermis, Azadirachta indica, Curcuma longa*, and *Achyranthes asper*a, were collected from a certified nursery based on their traditional medicinal use and availability. A qualified taxonomist in the Department of Botany authenticated the plant materials. Identification was confirmed using standard herbarium references and taxonomic keys described in relevant botanical literature. Voucher specimens of each plant species were deposited in the Herbarium of the Lanzhou Institute of Husbandry and Pharmaceutical Sciences for future (16).

The collected leaves were thoroughly washed under running tap water to remove dust and debris, then rinsed with distilled water. The plant materials were then air-dried under shade at room temperature for several days to preserve heat-sensitive phytoconstituents. After complete drying, the leaves were ground into a coarse powder using a sterile electric grinder. The powdered samples were stored in clean, airtight containers at room temperature until further extraction and phytochemical analysis.

### 2.4 Preparation of medicinal extraction procedure / determination of extract yield

The extraction of each plant material was carried out following previously established methods with slight modification (17). Four solvents of increasing polarity: petroleum ether, ethyl acetate, methanol, and finally distilled water were used to extract powdered samples of the plants in succession, as summarized in Tables 1, 2.

**Table 1:** Solvent extraction summary.

Extraction parameter	Methanol solvent	Notes
Solvent used	99% Methanol	Highly efficient polar solvent
Extraction method	Cold maceration (72 h)	Gentle, preserves heat-sensitive compounds
Solvent: sample ratio	500 ml: 50 g	Standard ratio for leaf extraction
Temperature range	Room temperature (25 °C −28 °C)	Prevents degradation of phytochemicals
Filtration method	Whatman no. 1 filter paper	Removes particulates
Concentration method	Rotary evaporation at 40 °C−45 °C	Prevents loss of active compounds
Storage of extract	4 °C in amber bottles	Protects from light and oxidation

**Table 2:** Extract yield of methanol leaf extracts.

Plant species	Fresh weight of leaves (g)	Dry weight of powder (g)	Amount of extract obtained (g)	% Yield (w/w)
*Aloe vera*	250	38	6.2	16.3%
*Lawsonia inermis*	250	42	7.8	18.6%
*Azadirachta indica*	250	40	8.4	21.0%
*Curcuma longa*	250	45	9.6	21.3%
*Achyranthes aspera*	250	36	5.7	15.8%

In this case, 300 g of dried and powdered plant species were wetened with 1.5 L of petroleum ether and put in a VWR DS 500 orbital shaker at room temperature and shaken overnight (72 h). The solution was filtered using Whatman No. 1 filter paper, and the residual was re-extracted two more times using fresh petroleum ether. The pooling of all the filtrates was done to get the petroleum ether extract.

The obtained marc was dried in the air and subjected to the sequential extraction using ethyl acetate, methanol, and sterile distilled water by following the same procedure used in petroleum ether. To obtain the crude extracts, organic solvents were evaporated through a rotary evaporator, then the semi-solid residues were dried in a water bath at 40 °C.

After full drying, the yield percentage of each extract was determined using the dry weight. All crude extracts were kept at 4 °C pending use. In case of biological assays, the dried methanol extracts of *Aloe vera* (L.), *Lawsonia inermis* (L.), *Azadirachta indica* (A. Juss.), Curcuma longa (L.), and *Achyranthes aspera* (L.) were dissolved in 10 percent DMSO and kept at 4 °C until the analysis.

### 2.5 Phytochemical screening of medicinal plants

The methanolic extracts of *Aloe vera, Lawsonia inermis, Azadirachta indica, Curcuma longa*, and *Achyranthes aspera* were analyzed for the presence of major bioactive compounds, including alkaloids, saponins, tannins, phenolics, flavonoids, and steroids, using standard qualitative procedures (18, 19). The presence of each phytochemical was recorded as positive (+) or strongly positive (++), and results are summarized in Table 4.

#### 2.5.1 Test for alkaloids

The tests were used to identify alkaloids: Mayer and Dragendorff. In the Mayer test, a test tube was filled with 2 ml of plant extract, and a small volume of Mayer reagent (a solution of potassium mercuric iodide) was added to it. The appearance of a creamy white or yellowish precipitate was a sign of the presence of alkaloids. In the test of Dragendorff, 2 ml of the plant extract was mixed with a few drops of the Dragendorff reagent (potassium bismuth iodide solution). The development of an orange or reddish-brown precipitate was taken to be positive for alkaloids (20).

#### 2.5.2 Test of saponins

Were identified with the mediums of the foam test and the hemolysis test. During the foam test, 510 ml of distilled water was put in a test tube, and 50 ml of the plant extract was added to it. The mixture was shaken vigorously (1–2 min). The appearance of a stable and persistent foam, which lasted several minutes, was a sign of the presence of saponins. When doing the hemolysis test, the plant extract was introduced into a suspension of red blood cells (e.g., goat or sheep blood). The presence of saponins was confirmed by the occurrence of hemolysis, which was detected by the change of color of the solution (21).

#### 2.5.3 Test for tannins and phenolics

Two qualitative tests were conducted to determine the presence of tannins and phenolics. In the case of the Ferric Chloride test, the plant extract in aqueous solution was 2 ml combined with 3 drops of 1% ferric chloride (FeCl 3) solution in a test tube. The mixture was monitored to attain blue, green, or black to detect the presence of the phenolic compounds or tannins.

#### 2.5.4 Test for flavonoids

The alkaline reagent test and the ammonium hydroxide test were used as tests to establish the presence of flavonoids. In the alkaline reagent test, the plant extract (2 ml) was put into five drops of an alkaline solution of sodium hydroxide (NaOH). It turned yellow, and on the addition of heavily diluted hydrochloric acid (HCl), it turned colorless, which showed that it had flavonoids. In a different test, a 10 percent ammonium hydroxide solution was put in a test tube with the plant extract. The presence of flavonoids was established by the yellow fluorescence (22).

#### 2.5.5 Shinoda test (magnesium and hydrochloric acid test)

Flavonoids were confirmed using the Shinoda test. A small amount of the plant extract was added to a test tube, followed by a few magnesium turnings and a few drops of concentrated hydrochloric acid (HCl). The development of a pink, red, or magenta coloration indicated the presence of flavonoids (23). For the Gelatin test, 5 ml of a 1% gelatin solution containing 0.85% sodium chloride was prepared. To this solution, 2 drops of the plant extract were added and mixed gently. The formation of a white precipitate indicated the presence of tannins (23).

#### 2.5.6 Test for steroids

The Salkowski test was used to determine the presence of steroids. In short, 5 ml of the aqueous plant extract was combined with 2 ml of chloroform in a test tube, and 2 ml of concentrated sulfuric acid (H 2 S O 4) was carefully added to the side of the tube. The mixture was left to rest for 5 min. The appearance of red color in the lower layer of chloroform was an indication of the presence of steroids. In another experiment, the plant extract was combined with 5 ml of chloroform, and then 500 μL of the plant extract and 5 ml of concentrated H 2 SO 4 were gradually added. The change of violet to blue or green was taken to be a positive result or the development of the blue-green ring at the interface. Also, the addition of fluorescence between the yellow and green colors of the upper layer further proved the presence of steroids when acid is added (20).

### 2.6 Bacterial culture and inoculum preparation

Fresh bacterial cultures were revived from frozen stocks and streaked onto Mueller-Hinton agar (MHA) plates, followed by incubation at 37 °C for 24 h. After 18–24 h of growth, a single well-isolated colony was selected and suspended in 3 ml of sterile saline. The suspension was vortexed to ensure homogeneity, and the turbidity was adjusted to match a 0.5 McFarland standard (approximately 1 × 10^∧^8 CFU/ml).

### 2.7 Antibacterial activity (agar well diffusion assay)

The antibacterial activity of the methanolic plant extracts was evaluated using the agar well diffusion method. Clinical bacterial isolates commonly associated with wound infections (*Staphylococcus aureus, Streptococcus agalactiae, Proteus mirabilis, Escherichia coli, Pseudomonas aeruginosa*, and *Klebsiella pneumoniae*) were tested alongside standard reference strains obtained from the National Referral Bacteriology and Mycology Laboratory. All isolates were subcultured on nutrient agar and subsequently grown in nutrient broth overnight. The turbidity of each culture was adjusted to a 0.5 McFarland standard (approximately 1 × 10^8^ CFU/ml) and uniformly spread onto Mueller–Hinton agar plates using sterile cotton swabs to produce a confluent bacterial lawn.

Wells measuring 6–8 mm in diameter were aseptically created using a sterile cork borer. Each well was filled with 50–100 μL of plant extract (100 mg/ml dissolved in 10% DMSO). Ciprofloxacin (10 μg/disc) served as the positive control, while 10% DMSO alone was used as the negative control; the solvent produced no inhibition zone, confirming the absence of antibacterial effects.

The plates were allowed to stand at room temperature for 20–30 min to permit pre-diffusion of the samples, then incubated in an inverted position at 37 °C for 18–24 h. Following incubation, antibacterial activity was assessed by measuring the diameter of the clear inhibition zones surrounding each well. Results were recorded in millimeters as the mean of triplicate measurements.

### 2.8 Interpretation of zone diameter and inhibition activity

This table describes the relationship between zone diameter (in millimeters) and inhibition activity in antimicrobial testing. “Not seen” indicates no observable inhibition zone. The inhibition activity is categorized as follows: (–) for no activity, (+) for weak activity (>10 mm), (++) for moderate activity (10–14 mm), (+++) for strong activity (15–19 mm), and (++++) for very strong activity (20–30 mm). Each phytochemical's presence or absence was denoted by a (–) and (+) in the results (Table 3).

**Table 3:** Zone diameters for assessing inhibitory activity (5).

Zone diameter	Inhibition activity
Not seen	(–)
>10	(+)
10–14	(++)
15–19	(+++)
20–30	(++++)

### 2.9 Statistical analysis

A graphical representation was created with Microsoft Office Excel 2007. The descriptive statistics of the variables under study were the frequency count and percentage analysis. Also, SPSS Statistics was used to analyze the data. The observed discrepancies were statistically tested by comparing the group means.

## 3 Result

### 3.1 Phytochemical constituents of methanol leaf extracts

**Figure 1:**
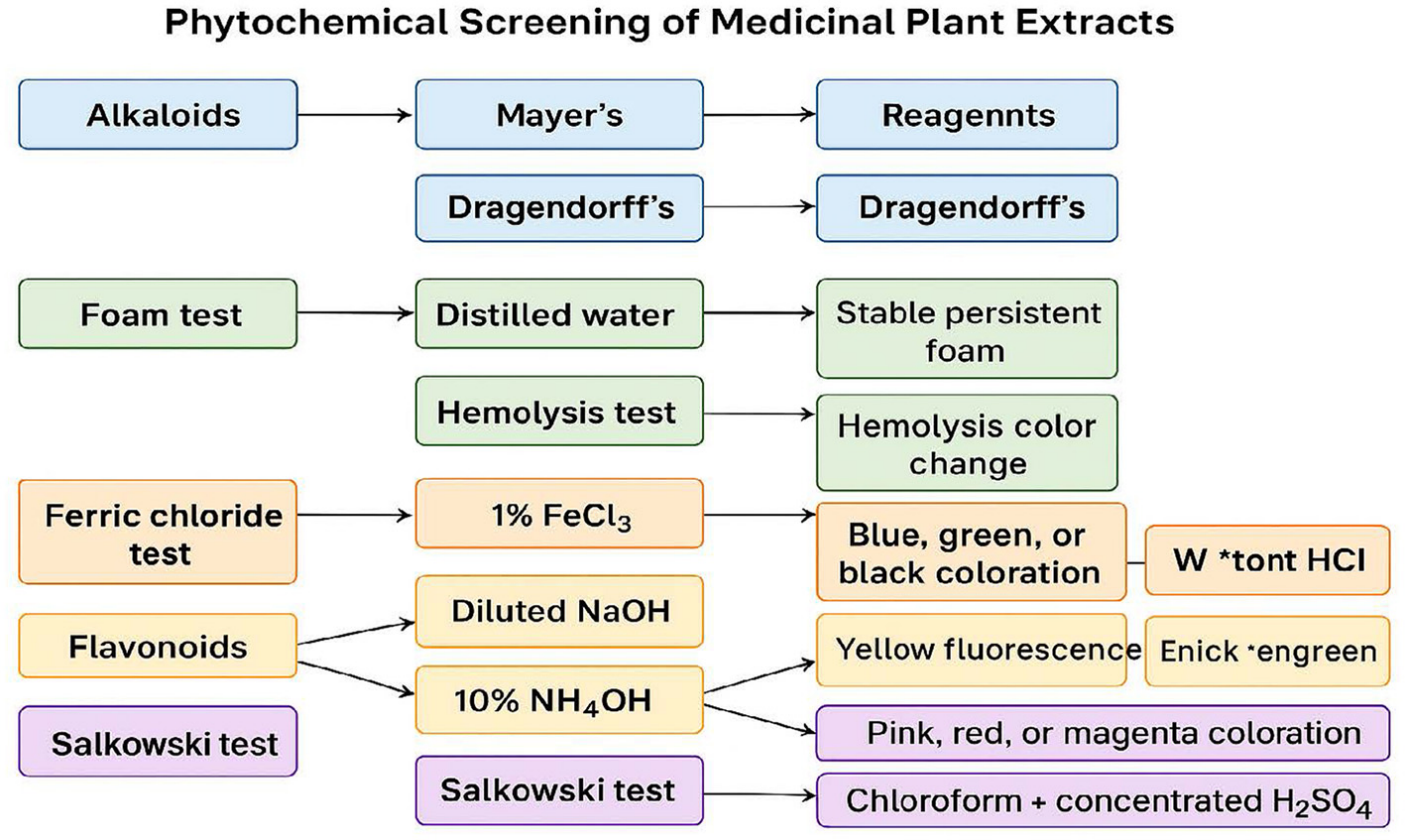
Flowchart of phytochemical screening of *Aloe vera, Lawsonia inermis, Azadirachta indica, Curcuma longa*, and *Achyranthes aspera* leaf extracts, showing tests, reagents, and positive results.

Qualitative phytochemical screening of the methanol extracts of *Aloe vera, Lawsonia inermis, Azadirachta indica, Curcuma longa*, and *Achyranthes aspera* revealed the presence of several bioactive secondary metabolites known for antimicrobial and wound-healing activities. Alkaloids, flavonoids, tannins, phenolics, saponins, and steroids were detected in all five extracts, although their intensity varied among species. *L. inermis* and *C. longa* exhibited strongly positive reactions for phenolics, tannins, and flavonoids, while *A. Indica* demonstrated a strong presence of alkaloids and saponins. The results are summarized in Table 4 and Figure 1.

**Table 4:** Phytochemical profile of methanol extracts of the selected medicinal plants.

Phytochemicals	*Aloe vera*	*L. inermis*	*A. indica*	*C. longa*	*A. aspera*
Alkaloids	+	**++**	**++**	**+**	**+**
Flavonoids	**+**	**++**	**+**	**++**	**+**
Tannins	**+**	**++**	**+**	**++**	**+**
Phenolics	**+**	**++**	**+**	**++**	**+**
Saponins	**+**	**+**	**++**	**+**	**+**
Steroids	**+**	**+**	**+**	**+**	**+**

+ = present; ++ = strongly present.

### 3.2 Bacterial isolate

A total of 123 wound samples were analyzed for bacterial wound infection. Table 5 presents the prevalence of the 123 bacterial isolates identified as the six major species. *Staphylococcus aureus* and *Streptococcus agalactiae* were the predominant gram-positive bacteria, found in 38 and 17 isolates, respectively. Among gram-negative bacteria, *P. mirabilis, E. coli, P. aeruginosa*, and *K. pneumoniae* were found in 22, 18, 15, and 13 isolates, respectively. Identification was based on morphology, cultural characteristics, and staining, and confirmed by biochemical tests.

**Table 5:** Prevalence of major bacterial isolates in wound samples (*n* = 123).

Bacterial species	Number of isolates	Percentage (%)
*Staphylococcus aureus*	38	30.9
*Streptococcus agalactiae*	17	13.8
*Proteus mirabilis*	22	17.9
*Escherichia coli*	18	14.6
*Pseudomonas aeruginosa*	15	12.2
*Klebsiella pneumoniae*	13	10.6
Total number	123	100

### 3.3 Antibacterial activity of methanol leaf extracts

**Figure 2:**
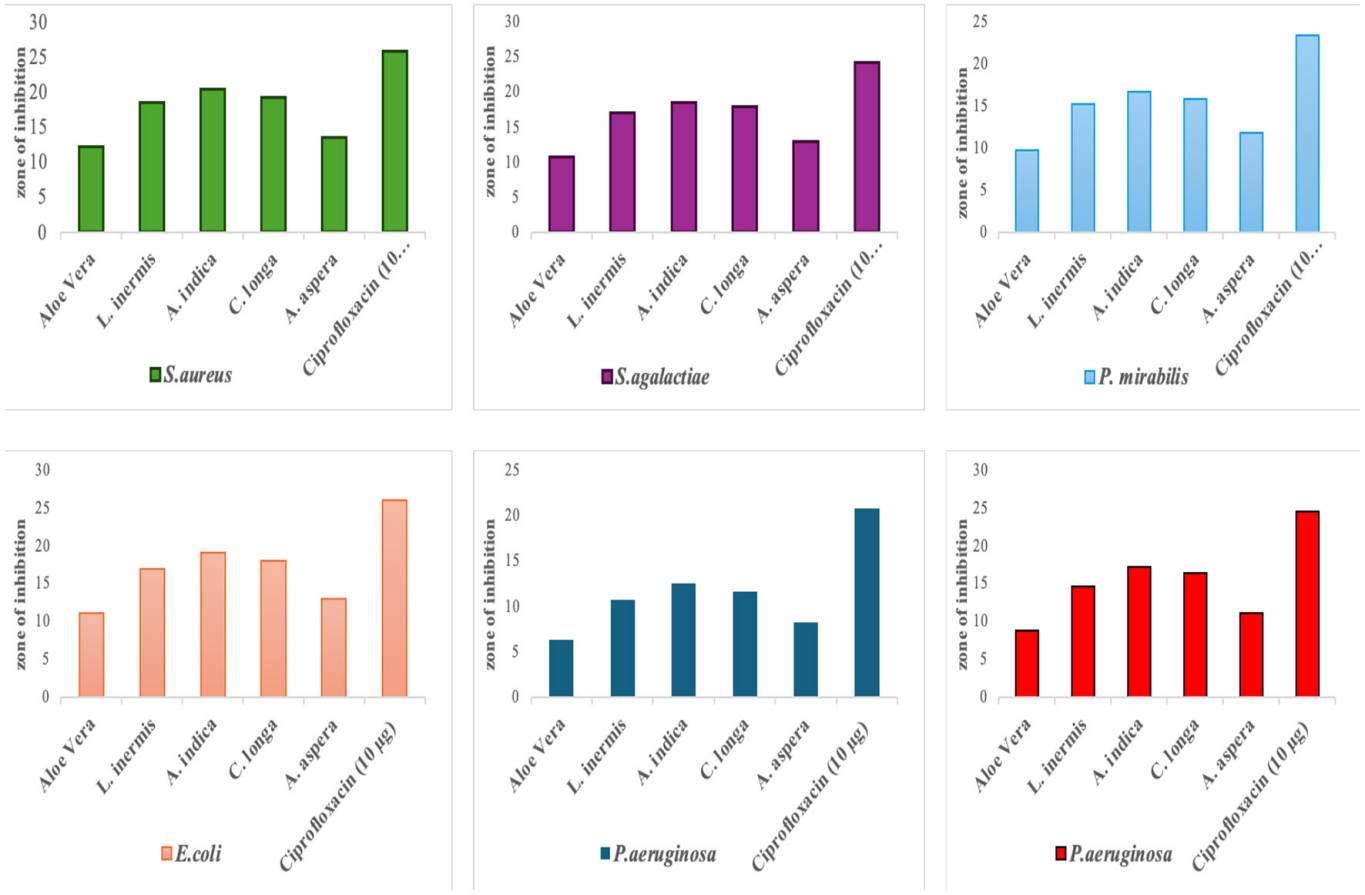
Antibacterial activity of methanolic leaf extracts against gram-positive and gram-negative bacterial isolates. The *Y*-axis represents the diameter of the inhibition zones in millimeters (mm).

The antibacterial activity of the methanolic plant extracts is presented in Table 6 and Figure 2. *Azadirachta indica* exhibited the highest inhibitory effects across all isolates, with zones of inhibition ranging from 12.5 ± 0.5 mm against *P. aeruginosa* to 20.4 ± 0.9 mm against *S. aureus*. *Curcuma longa* also demonstrated strong antibacterial activity, producing inhibition zones between 11.6 ± 0.5 mm and 19.2 ± 0.7 mm. Moderate inhibition was observed with *Lawsonia inermis* and *Achyranthes aspera*, while *Aloe vera* showed comparatively lower activity, especially against *Pseudomonas aeruginosa* (6.4 ± 0.3 mm).

**Table 6:** Antibacterial activity (zone of inhibition in mm) of methanol leaf extracts against clinical bacterial isolates.

Bacterial isolate	*Aloe vera*	*L. inerms*	*A. indica*	*C. longa*	*A. aspera*	Ciprofloxacin (10 μg)	Negative control
*S. aureus*	12.3 ± 0.6	18.5 ± 0.8	20.4 ± 0.9	19.2 ± 0.7	13.6 ± 0.5	25.8 ± 0.6	0
*S.agalactiae*	10.7 ± 0.5	17.1 ± 0.7	18.6 ± 0.6	17.9 ± 0.8	12.9 ± 0.4	24.3 ± 0.5	0
*P. mirabilis*	9.8 ± 0.4	15.2 ± 0.6	16.7 ± 0.5	15.9 ± 0.6	11.8 ± 0.4	23.4 ± 0.7	0
*E. coli*	11.2 ± 0.5	16.9 ± 0.6	19.1 ± 0.8	18.0 ± 0.7	13.1 ± 0.5	26.1 ± 0.4	0
*P.aeruginosa*	6.4 ± 0.3	10.8 ± 0.4	12.5 ± 0.5	11.6 ± 0.5	8.3 ± 0.3	20.8 ± 0.5	0
*K.pneumoniae*	8.9 ± 0.4	14.6 ± 0.5	17.3 ± 0.6	16.4 ± 0.6	11.2 ± 0.4	24.6 ± 0.6	0

Ciprofloxacin (10 μg), used as the positive control, produced the largest inhibition zones for all bacteria (20.8 ± 0.5 mm to 26.1 ± 0.4 mm), confirming its superior antibacterial potency. The negative control (10% DMSO) showed no inhibitory effect.

The comparative analysis presented in Table 7 highlights the relationship between phytochemical abundance and antibacterial efficacy among the five methanolic plant extracts. *Azadirachta indica* demonstrated the strongest overall antibacterial activity, which corresponds with its high content of alkaloids and saponins, producing the largest inhibition zones across all bacterial isolates. *Curcuma longa* also exhibited strong inhibitory effects, consistent with its rich content of phenolics, tannins, and flavonoids, which are known for their antimicrobial potency. *Lawsonia inermis* showed moderate antibacterial activity, aligning with its high levels of tannins and phenolic compounds. On the contrary, *Achyranthes aspera* exhibited moderate to weak inhibition, although it contained vital bioactive constituents. Aloe vera was the poorest in its antibacterial effect, especially against *Pseudomonas aeruginosa*, which indicates a relatively low concentration of highly active phytochemicals in the methanolic extract in it. Generally, the comparative findings show a definite relationship between phytochemical richness, particularly alkaloids, flavonoids, and phenolic compounds, and the intensity of antibacterial effects of the sampled medicinal plants.

**Table 7:** Comparative analysis of phytochemical richness and antibacterial performance of methanolic leaf extracts.

Plant species	Key phytochemicals strongly present	Highest zone of inhibition (mm)	Lowest zone of inhibition (mm)	Overall activity level	Most susceptible bacteria	Least susceptible bacteria
*Azadirachta indica (Neem)*	Alkaloids, Saponins	20.4 ± 0.9 (*S. aureus*)	12.5 ± 0.5 (*P. aeruginosa*)	Very strong (+++/++++)	*S. aureus, E. coli*	*P. aeruginosa*
*Curcuma longa (Turmeric)*	Phenolics, Tannins, Flavonoids	19.2 ± 0.7 (*S. aureus*)	11.6 ± 0.5 (*P. aeruginosa*)	Strong (+++)	*S. aureus, E. coli*	*P. aeruginosa*
*Lawsonia inermis (Henna)*	Phenolics, Tannins, Flavonoids	18.5 ± 0.8 (*S. aureus*)	10.8 ± 0.4 (*P. aeruginosa*)	Moderate (++)	*S. aureus, E. coli*	*P. aeruginosa*
*Achyranthes aspera*	Alkaloids, Phenolics	13.6 ± 0.5 (*S. aureus*)	8.3 ± 0.3 (*P. aeruginosa*)	Moderate to weak (+/++)	*S. aureus*	*P. aeruginosa*
*Aloe vera*	Saponins, Steroids	12.3 ± 0.6 (*S. aureus*)	6.4 ± 0.3 (*P. aeruginosa*)	Weak (+)	*S. aureus*	*P. aeruginosa*

## 4 Discussion

The current research paper assessed the antimicrobial effects of methanolic leaf extracts of *Aloe vera, Lawsonia inermis, Azadirachta indica, Curcuma longa*, and *Achyranthes* against six microbial pathogens that were most often linked to wound infections. The five medicinal plants were found to be inhibitory to different degrees, which justifies their cultural healing in treating wounds. In general, it was found that *A. indica* and *C. longa* had the most pronounced antibacterial activity, *L. inermis* and *A. aspera* demonstrated average performance, whereas *A. Vera* demonstrated relatively low levels of inhibitory activity. These results are in line with a number of previous reports that indicate that the extraction solvent and phytochemical profile of these species vary in terms of their antibacterial potential.

The antibacterial activity observed in the present study may be associated with the presence of various phytochemical compounds such as flavonoids, phenolics, alkaloids, tannins, and saponins identified in the methanolic extracts of the studied plants. These phytochemicals have been widely reported to possess antimicrobial properties against both Gram-positive and Gram-negative bacteria. However, the exact mechanism of antibacterial action was not investigated in the present study. Therefore, it cannot be conclusively stated that the antibacterial effect is due to bacterial cell membrane disruption. Further studies involving detailed mechanistic analyses, such as membrane permeability assays or leakage of intracellular components, are required to determine the precise mode of antibacterial action of these plant extracts (24).

Past studies are also supported by the antibacterial effect of *Curcuma longa. C. longa* in the present study had a powerful inhibition effect against *S. aureus* and *E. coli* in conformity with other studies conducted by (25, 26), who reported the anti-bacterial, antioxidant, and anti-inflammatory effects of curcumin and the associated phenolic compounds. This high antibacterial effect can be explained by the high phytochemical content of this plant in flavonoids and phenolics, which have been established to disrupt the microbial membranes, interfere with the energy metabolism, and inhibit nucleic acid production. These findings indicate that methanol is an effective solvent for extracting the curcumin-related metabolites that have a high antimicrobial potential.

The intermediate antibacterial activity recorded in this study with *Lawsonia inermis* is consistent with the results of (27, 28), who had reported inhibition of Gram-positive and Gram-negative bacteria by *L. inermis* extracts. In the present research, the plant was observed to be significantly active against *S. aureus* and *E. coli*, which confirms the hypothesis of lawsone, which is the predominant naphthoquinone of henna, contributing to the central role of its antimicrobial activity. The inhibition zones obtained are within the range that has been previously reported, indicating a uniform antimicrobial action, no matter what extraction method is used or what variations in bacterial strain occur.

On the same note, the antibacterial properties of *Achyranthes aspera* were moderate in our trials, with prior findings being reported. These scientists explained the antimicrobial properties of *A. aspera* by the presence of alkaloids, saponins, and phenolic phytochemicals, which were also found in the current study. The similarity in the inhibitory profiles of the studies is an indication that the plant has stable antibacterial properties that are consistent in various extraction conditions. *Aloe vera* had the lowest antibacterial effect when compared to the extracts that were tested, especially in relation to *Pseudomonas aeruginosa*. This finding correlates with various previous studies, such as that which noted that *Aloe* extracts tend to exhibit weak antibacterial action except when fresh gel or concentrated latex is utilized (29). The relatively low inhibition zones recorded in the present study may be attributed to a lower concentration of anthraquinones in the dried methanol extract compared to fresh gel preparations. However, the moderate activity observed against *S. aureus* agrees with (30), who observed that Aloe has greater inhibitory properties toward Gram-positive bacteria because they have a permeable cell wall architecture.

The testing of bacteria across all plants exhibited a trend of greater susceptibility of Gram-positive bacteria isolates as opposed to Gram-negative ones. This has been generally reported in phytochemical studies and is ascribed to structural features of Gram-negative bacteria that have an extra outer membrane composed of lipopolysaccharides, which are highly resistant to phytochemicals. The findings of the current research are similar to the findings of other researchers who reported less penetration of the plant extract in the Gram-negative cell envelope (31, 32).

The phytochemical analysis performed in this paper showed a high abundance of flavonoids, tannins, phenolics, saponins, and alkaloids, all of which are well-known antimicrobial agents. The high amount of such compounds in *A. indica* and *C. longa* can be directly connected to their good antibacterial activity. It has been demonstrated in previous studies that phenolics and flavonoids destabilize bacterial membranes, tannins precipitate bacterial proteins, alkaloids disrupt DNA replication, and saponins raise the permeability of membranes. The similarity in the phytochemical profile and antimicrobial activity is an indication that the two compounds work in harmony to increase antimicrobial activity.

In general, the results of this paper support the previous reports that there are bioactive compounds produced in medicinal plants that can be used to inhibit pathogenic bacteria related to wound infection. Interesting events of neem and turmeric, especially, point to the fact that they can be further improved into natural antimicrobial formulations. However, further studies such as minimum inhibitory concentration (MIC) and minimum bactericidal concentration (MBC), isolation of the compound, toxicity, and *in vivo* tests are required to determine their safety, potency, and therapeutic utility.

Another important aspect that requires further investigation is the safety profile of the plant extracts. Cytotoxicity studies, such as the MTT assay using mammalian cell lines, were not performed in the present study. Evaluation of cytotoxic effects is essential to determine the potential therapeutic safety of plant-derived antimicrobial agents. Therefore, future research should assess the cytotoxicity and biocompatibility of these extracts before considering their possible clinical applications.

## 5 Significance and future directions

The research has shown that the chosen medicinal plants, in particular, *Azadirachta indica and Curcuma longa*, have significant antibacterial effects against significant wound-infecting agents, which justifies the use of previously traditionally used medicinal plants and which also emphasizes them as possible natural, accessible substitutes for synthetic antibiotics. The availability of heterogeneous phytochemical groups also supports their therapeutic importance and speculates on possible synergistic effects that may be exploited to create plant-based wound-healing preparations.

The research area in the future must include defining MIC and MBC values, isolating and identifying the particular bioactive compounds of the antibacterial action, and a comparative study of plant extracts with different solvents to increase the diversity of compounds. To determine safety and clinical relevance, *in vivo* experiments, such as wound-healing models and cytotoxicity tests, are necessary. Also, comparisons with multidrug-resistant strains and the determination of how these extracts can be incorporated into gels, ointments, or nanoparticle-based delivery systems would cement their potential with regard to their use in the real world.

## 6 Conclusion

The current research showed that the *Azadirachta indica, Curcuma longa, Lawsonia inermis, Achyranthes aspera*, and *Aloe vera* leaf extracts in methanol have different levels of antibacterial effect against pathogens associated with wounds. *A. indica* and *C. longa* had the greatest inhibitory activities in all the bacterial isolates, which was in agreement with their high level of bioactive phytochemicals, including phenolics, flavonoids, alkaloids, and saponins. The activity of *L. inermis* and *A. aspera* was moderate, and *A. Vera* had relatively weak inhibition, especially when targeting *Pseudomonas aeruginosa*. The Gram-negative species were usually more resistant than the Gram-positive due to known differences in cell wall structure and permeability.

All in all, the findings confirm the conventional use of the mentioned medicinal plants in wound management and suggest that they may serve as sources of natural antimicrobials. The fact that *A. indica* and *C. longa* are highly active indicates that they can be good prospective sources of production of plant-based therapeutic formulations. Nevertheless, more research is required to determine MIC and MBC, isolate active compounds, evaluate their toxicity, and conduct *in vivo* testing to confirm their safety, maximize their efficiency, and adapt them to contemporary clinical practice.

## Data Availability

The original contributions presented in the study are included in the article/supplementary material, further inquiries can be directed to the corresponding authors.
